# Complete Laceration of Right Arm

**Published:** 1889-06

**Authors:** Rudolph Menger

**Affiliations:** San Antonio, Texas


### COMPLETE LACERATION OF RIGHT ARM.

BY DR. RUDOLPH MENGER, SAN ANTONIO, TEXAS.

[For Daniel's Texas Medical Journal.]

ANTON ROATZSCH, a young engineer, aged 25 years, of robust stature, entangled his shirt sleeve and right hand and arm in a coffee-rolling machine whilst repairing the leather strap, which had come off of one of the twisting wheels. The machine was running at the time at full speed. In order to reach the twisting wheel the man stod on a lage box near by and below it. But, before he knew how it happened,' his sleeve and arm were caught immediately between the broad leather strap and the machinery above him, and he was lifted several times, about four feet from the ground,—until his arm gave way, severing it completely about two or two inches below the elbow joint, after which he fell to the ground.

In spite of the severe shock he had nerve enough to go to the engine, stop the same with his left hand from running and ordered a fellow-mate, who was horror stricken, to pick up his arm from the ground and bring it along with him to the next drug store, where he went on foot. In this condition I found the man sitting in the drug store, he being under the impression that his arm could be "sewed on" again. He was bleeding, as usual in such cases, but little from the lacerated parts, but his clothing was saturated with blood. The extensor muscles, the median nerve, and both radius and ulna were terribly lacerated, and, on examining the upper arm, a complete fracture of the humerus near the shoulder was revealed.

After giving some stimulants, etc., and applying an Esmarch's bandage, the man was removed to his house, as he objected to being taken to a hospital.

Fearing severe laceration and contusion of the elbow joint, muscles, nerves and aponeurosis, I amputated his arm above the elbow joint, just below the fracture of the humerus, kindly assisted by Dr. Berry. I was particularly careful in ligating the vessels and closing the stump, but although he stood the amputation well, unfortunately some after-hemorrhage set in about three hours after the operation. Assisted by Dr. Shropshire, we opened the stump and found it completely filled with clots. After their romoval we soon checked the oozing with hot water sponging, leaving the stump open and dressing it with iodoform and carbolated cotton. The next day the man, under existing circumstances, was restless, and on dressing the stump there was again some bleedin g from a very small muscular artery, which was ligated. From now on the man did remarkably well—until the fourth day when I found him very restless and the stump somewhat discolored, but he did not complain or his stump. Toward noon I was hurriedly summoned, and on arriving found him laboring under symptoms of traumatic tetanus. We tried all the usual remedies, bat he died at about 7 p. m. the same day.

On examining the part of the arm torn away by the machinery it was found that the radial, ulnar, and especially the median nerve were torn out several inches from the line of laceration, and the radius was dislocated, the arm having been considerably twisted during the time the man was caught and lifted by the machinery.

